# Hemobilia in the setting of cystic artery pseudoaneurysm secondary to type I Mirizzi syndrome

**DOI:** 10.1007/s12328-023-01806-w

**Published:** 2023-05-02

**Authors:** Thomas Williams, Adrian Maher, Kendal Redmond, Shinn Yeung, Bong Suk Ko

**Affiliations:** 1 Department of Gastroenterology and Hepatology, Princess Alexandra Hospital, 199 Ipswich Road, Woolloongabba, Brisbane, QLD 4102 Australia; 2 Faculty of Medicine, University of Queensland, Brisbane, Australia; 3 Department of Medical Imaging, Princess Alexandra Hospital, Brisbane, Australia; 4 Department of Hepatobiliary Surgery, Princess Alexandra Hospital, Brisbane, Australia

**Keywords:** Hemobilia, Mirizzi syndrome, Aneurysm, False, Cholangiopancreatography, Endoscopic retrograde

## Abstract

Hemobilia is an uncommon diagnosis and is often not suspected in the absence of recent hepatobiliary intervention or trauma. Hemobilia in the setting of cystic artery pseudoaneurysm secondary to type I Mirizzi syndrome is a rare occurrence. We report the case of a 61-year-old male who presented with epigastric pain and vomiting. Blood tests demonstrated hyperbilirubinemia with elevated inflammatory markers. Magnetic resonance cholangiopancreatography revealed type I Mirizzi syndrome in the presence of a 21 mm cystic duct stone. During endoscopic retrograde cholangiopancreatography, hemobilia was identified. Subsequent triple phase computed tomography imaging identified a 12 mm cystic artery pseudoaneurysm. Angiography with successful coiling of the cystic artery was accomplished. Cholecystectomy was performed, confirming type I Mirizzi syndrome. This case demonstrates the importance of considering ruptured pseudoaneurysm in patients presenting with evidence of upper gastrointestinal bleeding in the setting of biliary stone disease. Transarterial embolization, followed by surgical management, is effective in both the diagnosis and management of ruptured cystic artery pseudoaneurysm with associated hemobilia.

## Introduction

Hemobilia is an uncommon diagnosis defined by the extravasation of blood within the hepatobiliary tree [1]. The expanded role of hepatobiliary intervention over recent decades has led to the predominant etiology for hemobilia being iatrogenic, accounting for up to 65% of cases [1, 2]. Although less common, traumatic, malignant, inflammatory, infectious, and vascular processes can also be responsible for this finding [1–3]. Identifying and diagnosing hemobilia can be difficult, particularly if suspicion is low due to the absence of recent hepatobiliary intervention or trauma.

Mirizzi syndrome involves the compression of the bile duct secondary to an impacted stone at the gallbladder neck or cystic duct [4]. This condition is categorised according to the absence (type I) or presence (type II to V) of cholecystobiliary fistula formation secondary to the associated pressure and inflammation of the impacted stone [4]. Further complications of Mirizzi syndrome can include the development of biliary stricture or cholecystoenteric fistula. Pseudoaneurysm formation is rare, particularly involving the cystic artery, with only three reported cases [5–7].

Here we present the case of a 61-year-old male with hemobilia in the setting of a cystic artery pseudoaneurysm that developed secondary to type I Mirizzi syndrome. This is a rare finding, however, should be considered when there is evidence of hemobilia or upper gastrointestinal bleeding in the presence of biliary stones.

### Case report

A 61-year-old, obese (body mass index of 37.3 kg/m^2^) male presented to the emergency department with acute onset epigastric pain and vomiting. His medical history included a previous episode of cholecystitis one year prior, treated with antibiotic therapy with the patient declining a cholecystectomy at this time. There was no evidence of pseudoaneurysm on intravenous iodinated Contrast Enhanced Computed Tomography performed during this presentation. He did not have any other medical comorbidities, nor any previous surgical history.

At presentation, his temperature was 36.6 degrees Celsius, blood pressure 136/78 mmHg, and pulse rate 72 beats per minute. Physical examination elicited right upper quadrant abdominal tenderness with positive Murphy’s sign and no peritonism. The total bilirubin was 43 µmol/L, alkaline phosphatase 162 U/L, gamma-glutamyl transferase 648 U/L, alanine transaminase 227 U/L and aspartate transaminase 242 U/L. His hemoglobin was 138 g/L with a white cell count 14.2 × 10^9^/L. Intravenous iodinated Contrast Enhanced Computed Tomography demonstrated an amorphous, thickened gallbladder and surrounding oedema, with thickening of the hepatic duct. The common bile duct measured 5 mm in maximal diameter and the pancreas was unremarkable in appearance.

**Fig. 1:**
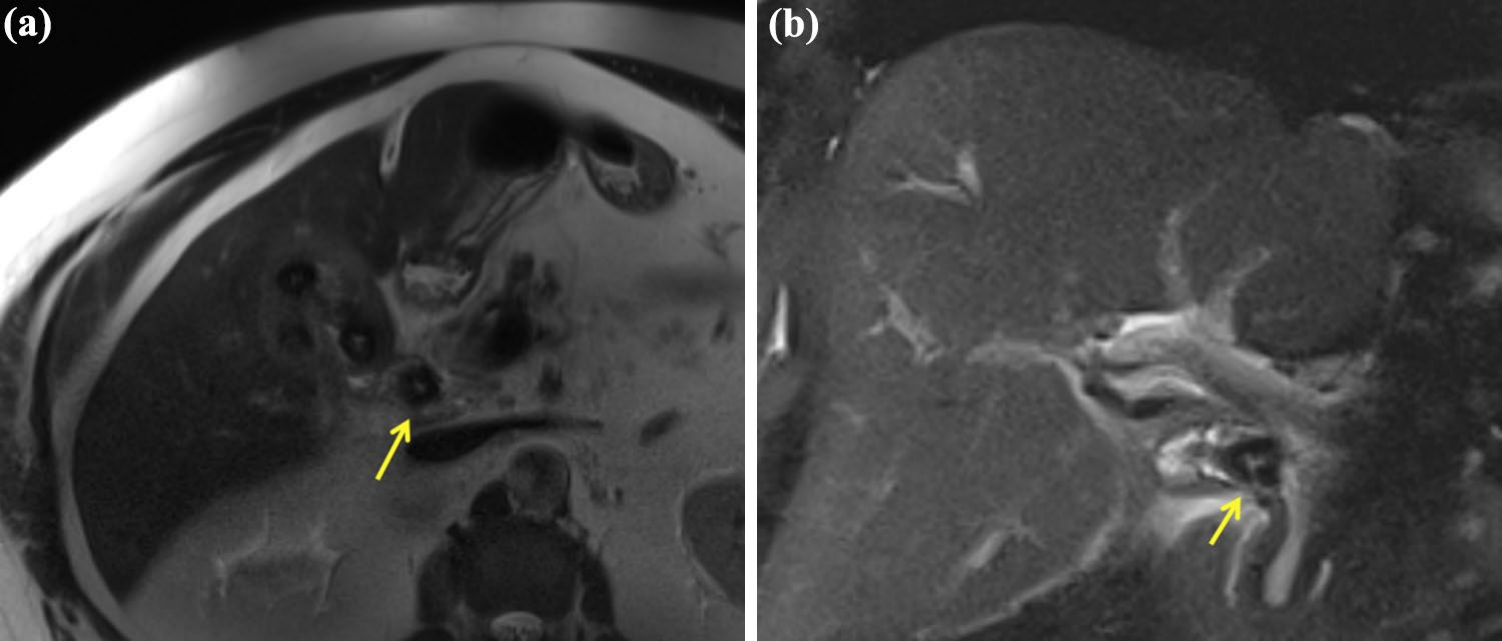
Magnetic resonance cholangiopancreatography imaging (T2 HASTE sequence) in the axial **a** and coronal **b** planes demonstrating cystic duct stone (yellow arrow) with associated common hepatic duct narrowing

Over the following 24 h the bilirubin incremented to 80 µmol/L with white cell count 22.5 × 10^9^/L and C-reactive protein 167 mg/L. His hemoglobin was unchanged. Magnetic resonance cholangiopancreatography (MRCP) demonstrated a thick-walled gallbladder with numerous cholelithiasis. A 21 mm stone was observed in the cystic duct, with associated oedema (Fig. 1). Adjacent common hepatic duct narrowing with proximal distension suggested type I Mirizzi syndrome, with no evidence of cholecystobiliary fistula formation. The patient was transferred to our quaternary centre for endoscopic retrograde cholangiopancreatography (ERCP).

**Fig. 2:**
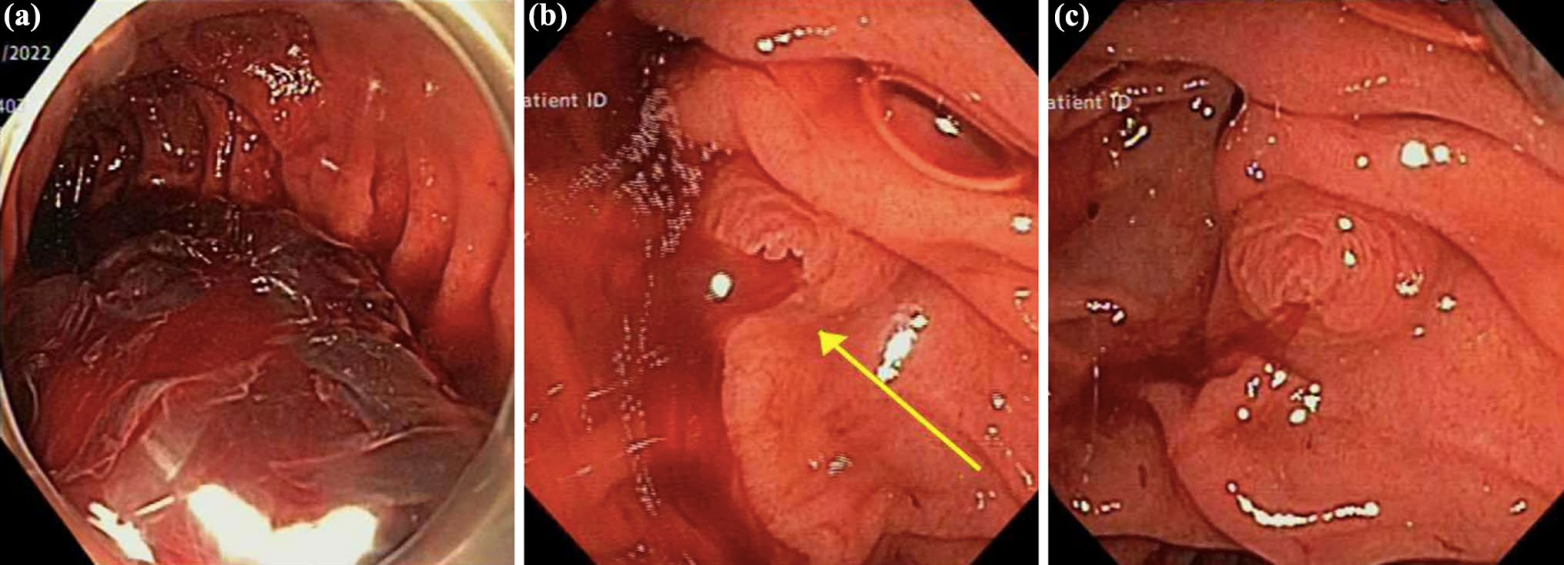
Image of endoscopic procedure performed demonstrating blood clot within the duodenum with forward viewing endoscope **a**. Blood visualised flowing from the major papilla (yellow arrow) on re-insertion of the duodenoscope (**b** and **c**)

The duodenoscope (TJF-Q190V Duodenoscope ®; Olympus, Tokyo, Japan) was advanced to the bulb of the duodenum and a significant volume of clotted blood was encountered. The duodenoscope was exchanged to a forward viewing endoscope (GIF-H180®; Olympus, Tokyo, Japan) to facilitate diagnostic assessment. There was a significant volume of clotted blood throughout the duodenum (Fig. 2a) which was irrigated and suctioned. The oesophagus, stomach, and the entire duodenum was inspected, with no visible lesion identified. The duodenoscope was reinserted and hemobilia was confirmed, with fresh blood hemorrhaging from the papilla (Fig. 2b, c).

**Fig. 3:**
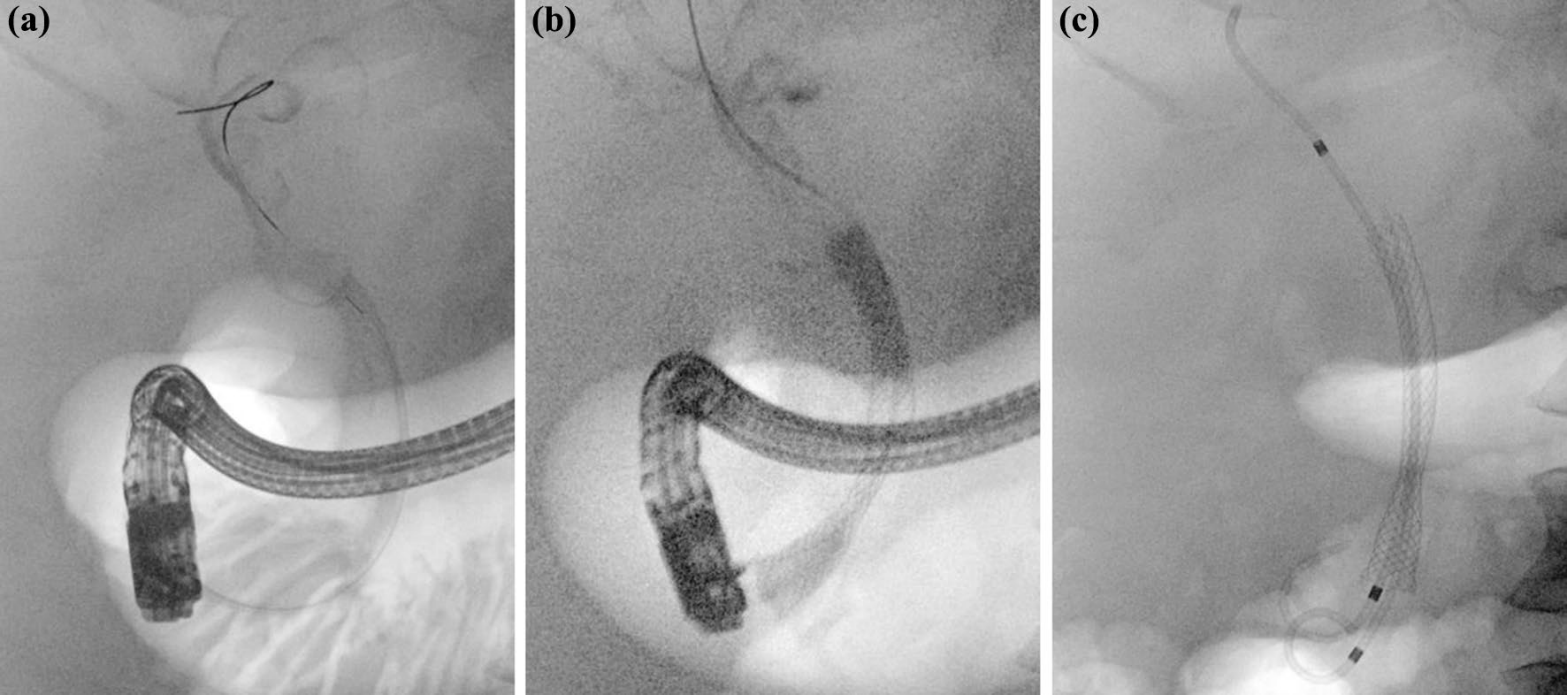
Initial ERCP performed for the management of suspected Mirizzi syndrome. The cholangiogram suggested extrinsic compression of the proximal bile duct with biliary lumen defects secondary to blood clots **a**. A fully covered metal stent was inserted, which did not traverse the extrinsic compression **b**. A plastic stent was inserted to traverse the narrowing and facilitate biliary drainage **c**

The papilla was cannulated with a wire (VisiGlide®; Olympus, Tokyo, Japan) and sphincterotome (CleverCut3V®; Olympus, Tokyo, Japan) was advanced. Contrast was injected and demonstrated a narrow common bile duct, with dilated intrahepatic ducts with extrinsic compression of the common hepatic duct, consistent with Mirizzi Syndrome type I (Fig. 3a). There was no contrast extravasation to suggest a bile leak. A sphincterotomy was performed, and a balloon trawl of the bile duct removed further blood clots. The cholangiogram did not improve following the balloon trawl. Based on the MRCP findings and cholangiogram suggesting external compression of the common hepatic duct, it was felt that the obstructive jaundice was likely secondary to compression by the cystic duct stone rather than blood clot.

A fully covered metal stent (Wallflex®; Boston Scientific, Marlborough, MA, USA) was initially inserted to achieve hemostasis, however, was unable to be advanced proximal to the obstruction due to the severity of the extrinsic compression (Fig. 3b). It was deployed distal to the obstruction, with ongoing hemobilia. A 7Fr 10 cm plastic stent (Advanix ®; Boston Scientific, Marlborough, MA, USA) was deployed, with the proximal end of the stent traversing the obstruction (Fig. 3c). Bile and blood flowed through the stent.

**Fig. 4:**
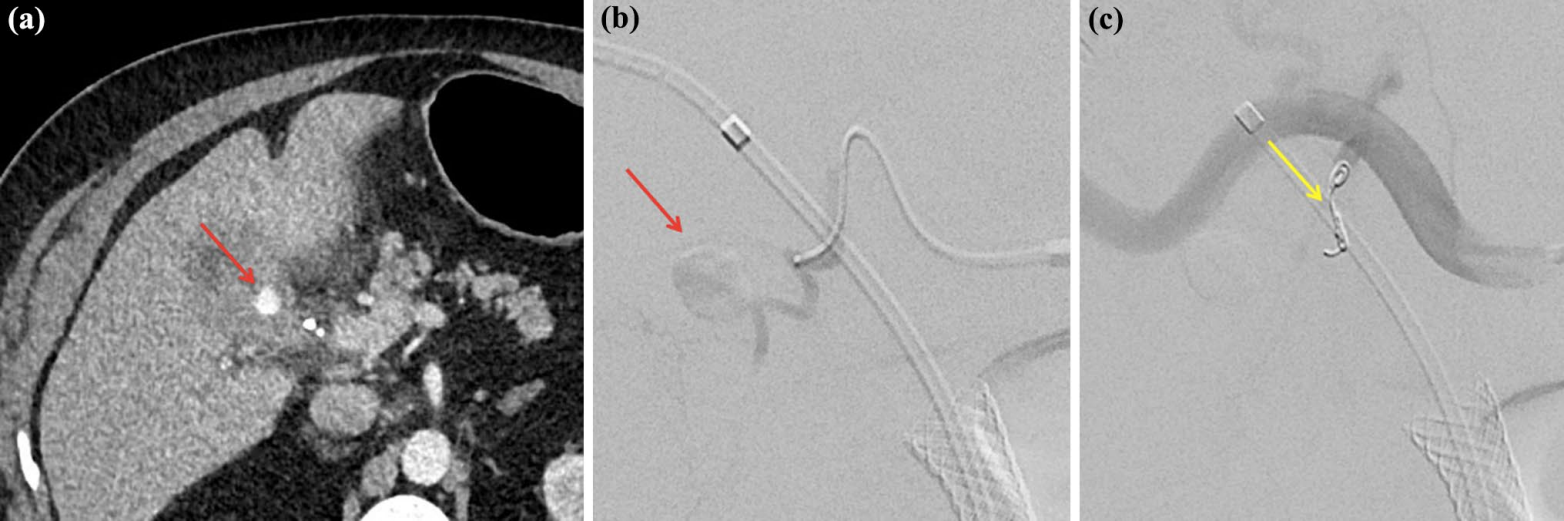
Arterial phase of triple phase contrast enhanced computed tomography in axial plane demonstrating arterial hyperenhancement (red arrow) **a**. Cystic artery pseudoaneurysm (red arrow) confirmed on angiography **b** with subsequent deployment of coil (yellow arrow) with successful embolization **c**

An urgent triple-phase liver computed tomography was arranged. This demonstrated a 12 mm focus of arterial hyperenhancement within the region of the medial gallbladder in close proximity to the cystic artery (Fig. 4a). Gross thickening, irregularity and enhancement of the gallbladder was evident, suggestive of chronic cholecystitis. There was no evidence of active bleeding at the time of this imaging. Following discussion with the hepatobiliary surgical department, angiography was performed with the aim to definitively diagnose and treat the possible pseudoaneurysm. At angiography, a cystic artery pseudoaneurysm was identified and successfully coiled. No further pseudoaneurysm was evident (Fig. 4b, c).

Cholecystectomy was arranged 48 h following embolization. The procedure was converted to open cholecystectomy to enable removal of multiple impacted gallstones within the gallbladder. A further stone impacted at the cystic duct and common bile duct junction was removed. Histopathological assessment of the gallbladder confirmed acute on chronic cholecystitis with no evidence of dysplasia or malignancy.

**Fig. 5:**
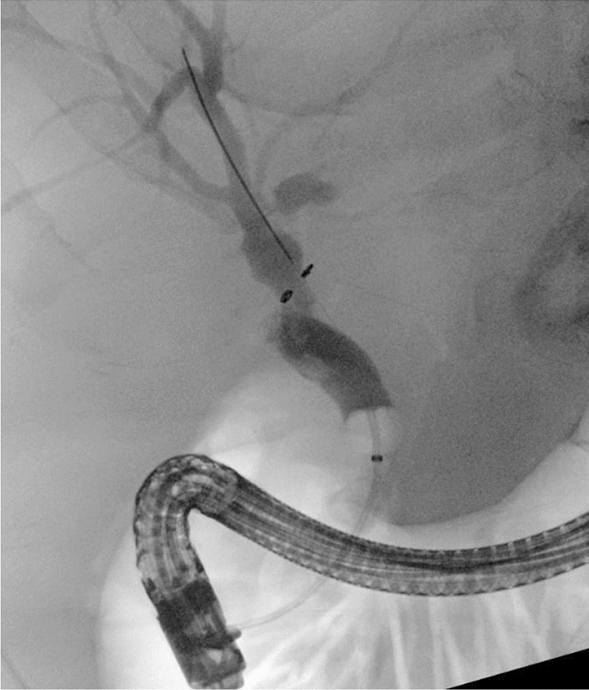
Cholangiogram at ERCP performed following cholecystectomy with stricture of common biliary duct

Following surgery, the patient continued to have improvement in their bilirubin and inflammatory markers. A repeat ERCP was performed which demonstrated an improved but persistent biliary stenosis in the main duct (Fig. 5). This was traversed with a 9-12 mm balloon and the patient did not require repeat biliary stenting. Repeat blood tests demonstrated return to premorbid liver enzyme levels.

## Discussion

We report a rare case of type I Mirizzi syndrome leading to the formation of a cystic artery pseudoaneurysm with associated hemobilia. Pseudoaneurysm formation occurs secondary to injury to the blood vessel wall with subsequent extravasation and containment in surrounding tissue. This may occur following trauma, medical intervention, infection, malignancy, or inflammation that damages the vessel wall [8].

Pseudoaneurysm is an uncommon cause for hemobilia, which is most commonly the result of prior hepatobiliary intervention, trauma or malignancy [1]. Within the rare cases of cystic artery pseudoaneurysms reported, the predominant etiologies are cholecystitis, followed by post-cholecystectomy, idiopathic and pancreatitis [9]. Direct iatrogenic vessel injury or inflammation with associated endothelial damage drive the weakening of the cystic artery with subsequent pseudoaneurysm formation [9].

Mirizzi syndrome is present in up to 5.7% of cholecystectomy cases [10], with type I Mirizzi syndrome accounting for 11% of cases [11]. To our knowledge, cystic artery pseudoaneurysm in the setting of Mirizzi syndrome has been rarely reported [5–7]. Suzuki et al. [5] reported an unruptured cystic artery pseudoaneurysm detected on cross-sectional imaging, while the other reported cases were associated with bleeding into the hepatobiliary tract [6, 7].

Our patient was diagnosed with a gallstone impacted within the cystic duct on MRCP with associated hepatic duct obstruction and no cholecystobiliary fistula. Prior to ERCP, there was no clinical symptoms or signs of upper gastrointestinal bleeding or change in hemoglobin. Direct mechanical injury, in addition to inflammation from the impacted stone and the associated cholecystitis, likely resulted in disruption to the cystic artery wall with extravasation into the hepatobiliary tract. While the source of hemobilia can originate at different locations within the biliary tree, it is felt that the cystic artery pseudoaneurysm was the source of hemobilia within this patient. This is suggested from the persistent hemobilia despite placement of the covered metal stent and cessation following angiography and coiling of the pseudoaneurysm.

Prior to operative management of Mirizzi syndrome and cholecystitis, transarterial embolization (TAE) is useful for the definitive diagnosis and treatment of pseudoaneurysm [1, 6, 7, 12]. TAE enables prevention of complications associated with visceral artery pseudoaneurysm formation. Following the diagnosis of hemobilia at ERCP within our case, successful coiling of the cystic artery pseudoaneurysm was performed prior to subsequent operative management.

At repeat ERCP, the cholangiogram demonstrated persistent narrowing of the bile duct following removal of the covered metal stent. This did not require intervention and the patient had improvement in liver enzyme tests. This stricture of the bile duct may have been associated with the embolization procedure performed for management of the pseudoaneurysm.

This case describes a ruptured cystic artery pseudoaneurysm associated with type I Mirizzi syndrome and hemobilia evident at ERCP. Successful endovascular coiling of the cystic artery pseudoaneurysm followed by operative management enabled effective treatment in the absence of acute postoperative complications. While rare, ruptured pseudoaneurysm is important to consider in the presence of symptoms and signs of upper gastrointestinal bleeding and gallstone disease.
